# Accelerometer-Derived Physical Activity, Sedentary Behavior, and Continuous Glucose Monitoring-Derived Glycemic Variability in Adults with Type 2 Diabetes: A Longitudinal Repeated-Measures Study

**DOI:** 10.3390/jfmk11030338

**Published:** 2026-08-28

**Authors:** Paul César Velásquez Porras, Alicia Olinda Neyra Aranda, Dimna Zoila Alfaro Quezada, Digmer Pablo Riquez Livia, Henri Emmanuel Lopez Gomez, Roberto Carlos Dávila-Morán

**Affiliations:** 1 Faculty of Health Sciences, Universidad César Vallejo, Lima 15314, Peru; pvelasquezp@ucvvirtual.edu.pe (P.C.V.P.); aneyraa@ucvvirtual.edu.pe (A.O.N.A.); 2 Faculty of Health Sciences, Universidad Privada San Juan Bautista, Lima 15067, Peru; dimna.alfaro@upsjb.edu.pe; 3 Faculty of Education and Physical Culture, Universidad Nacional de Educación Enrique Guzmán y Valle, Lima 15472, Peru; driquez@une.edu.pe; 4 Campus Huancayo, Universidad Tecnológica del Perú, Huancayo 12002, Peru; 5 Independent Researcher, Lima 15401, Peru; u26214220@utp.edu.pe

**Keywords:** time in range, wearable sensors, isotemporal substitution, free-living monitoring, within-person variation

## Abstract

**Background:** Day-to-day movement behavior may influence short-term glycemic variability in adults with type 2 diabetes, but free-living evidence integrating accelerometry with continuous glucose monitoring (CGM) remains limited. **Objectives:** This study examined whether daily moderate-to-vigorous physical activity (MVPA) and accelerometer-defined sedentary time were associated with 24-h within-day glycemic variability. **Methods**: In this longitudinal repeated-measures study, 140 adults with type 2 diabetes underwent 14 days of concurrent hip-worn ActiGraph GT3X+ accelerometry and FreeStyle Libre 2 CGM under free-living conditions in Lima, Peru. The primary outcome was a daily CGM-derived coefficient of variation (CV). Linear mixed-effects models separated within-person and between-person components, with Holm adjustment for the two primary within-person associations. **Results**: The fully adjusted primary analysis included 132 participants and 1728 participant-days. Higher within-person MVPA was associated with a lower daily CV (β = −0.27 percentage points per 10 min/day, 95% CI −0.45 to −0.09; Holm-adjusted *p* = 0.006), whereas greater accelerometer-defined sedentary time was associated with a higher CV (β = 0.05, 95% CI 0.01 to 0.09; Holm-adjusted *p* = 0.014). Secondary concurrent analyses showed favorable MVPA and opposite sedentary time associations for time in range, time above range, mean glucose, and glucose standard deviation (all false discovery rate *q* ≤ 0.018), with no statistically detectable association with time below range. Lagged associations were directionally consistent, but none remained statistically significant after multiplicity correction. **Conclusions**: Daily MVPA and accelerometer-defined sedentary times were associated with modest differences in within-day glycemic variability and concurrent CGM profiles. These observational findings do not establish causality or a specific exercise dose.

## 1. Introduction

Type 2 diabetes requires sustained, individualized management that integrates glycemic control with the assessment and management of diabetes-related complications and comorbidities, alongside ongoing health-related self-management behaviors [1,2]. Physical activity is a key component of this management because skeletal muscle contraction promotes glucose uptake and regular activity improves insulin sensitivity [3,4]. Because physical activity varies from day to day, its short-term relationship with glycemia may be relevant beyond weekly activity targets.

Sedentary behavior is a distinct dimension of movement behavior, defined by low energy expenditure while sitting, reclining, or lying [5]. In adults with type 2 diabetes, greater sedentary time and interruptions to sedentary time have been associated with CGM-derived glucose outcomes [6], while experimental work has shown that replacing prolonged sitting with standing and light-intensity walking can reduce 24 h glucose exposure [7]. Because hip-worn count-based accelerometry does not determine posture, the present study uses the term “accelerometer-defined sedentary time” for low-movement periods identified from accelerometer counts.

Continuous glucose monitoring (CGM) captures short-term glycemic patterns not reflected by glycated hemoglobin, including the time in range, time above range, time below range, mean glucose, and glycemic variability [8,9]. The coefficient of variation (CV), which expresses a glucose standard deviation relative to the mean glucose, was selected as the primary glycemic outcome because the study specifically focused on day-to-day variations in within-day glycemic variability rather than absolute glycemic exposure. By scaling glucose dispersion to the corresponding mean glucose level, a CV provides a relative measure of variability, whereas the mean glucose primarily reflects the glycemic level, and TIR, TAR, and TBR describe the distribution of time across clinically defined glucose ranges. These measures were therefore considered complementary secondary outcomes. Because multi-day monitoring improves the reliability of participant-level CV estimates [10], a daily CV was treated in the present repeated-measures framework as a day-level measure of within-day glycemic variability rather than as a stable measure of habitual glycemic variability.

Free-living evidence combining objective movement assessment with CGM in adults with type 2 diabetes remains limited and methodologically heterogeneous. Among the studies identified, four involved adults with type 2 diabetes. Paing et al. studied 37 adults using activPAL and CGM for up to 14 days; although daily movement and 24 h glucose measures were collected concurrently, the primary analyses related participant-level average sedentary time and sedentary breaks to average glycemic outcomes rather than modeling repeated day-level associations [6]. Gauthier et al. analyzed 28 insulin-pump-treated adults monitored for 7 days and reported both monitoring-period associations and a same-day comparison in which days with at least 1 h of physical activity above 1.5 METs were associated with lower glycemic variability [11]. Lee et al. studied 122 insulin-treated adults using Fitbit and CGM for 10 days and related behavioral factors, including daytime step count, to CGM metrics summarized over the monitoring period [12]. A subsequent study of 122 adults using 10 days of concurrent Fitbit and CGM data examined time-of-day-specific activity and circadian characteristics in relation to glycemic outcomes using cross-sectional association analyses [13]. In adults without diabetes, El Fatouhi et al. studied 85 participants over 2 weeks and explicitly evaluated both same-day and subsequent-day associations between daily step count and CGM-derived indices [14]. None of these studies explicitly decomposed repeated movement exposures into within-person and between-person components. Thus, evidence from repeated day-level analyses in adults with type 2 diabetes that jointly evaluate MVPA and accelerometer-defined sedentary time while separating within-person variations from between-person differences remains limited.

The present study examined day-to-day associations between accelerometer-derived movement behaviors and CGM-derived glycemic outcomes during 14 days of concurrent free-living monitoring in adults with type 2 diabetes. The primary objective was to determine whether daily MVPA and accelerometer-defined sedentary time were associated with 24 h within-day glycemic variability, assessed using a daily CGM-derived CV. We hypothesized that higher-than-usual MVPA would be associated with a lower daily CV, whereas greater-than-usual accelerometer-defined sedentary time would be associated with a higher daily CV. Secondary objectives were to examine concurrent associations with time in range, time above range, time below range, mean glucose, and glucose standard deviation; daily step count, light physical activity, accelerometer-defined sedentary breaks, and lagged analyses were exploratory.

## 2. Materials and Methods

### 2.1. Study Design and Setting

This observational longitudinal repeated-measures study was conducted among adults with type 2 diabetes attending the outpatient endocrinology service of a tertiary referral hospital in Lima, Peru. Concurrent accelerometer and continuous glucose monitoring (CGM) data were collected on repeated participant-days between January and December 2025 to examine day-to-day associations between movement behaviors and CGM-derived glycemic outcomes.

### 2.2. Participants

The source population comprised adults with an established clinical diagnosis of type 2 diabetes attending the outpatient endocrinology and diabetes clinic of the participating tertiary referral hospital in Lima, Peru. Participants were recruited consecutively using non-probability sampling. At baseline, trained research personnel reviewed the available clinical documentation and study eligibility criteria, confirmed eligibility, and then obtained written informed consent before any study procedures were initiated.

Eligibility criteria included age ≥ 18 years; an established clinical diagnosis of type 2 diabetes documented in the medical record before study enrollment and verified during the baseline eligibility review; ability to follow study procedures; and availability for concurrent accelerometer and CGM. Diabetes type was based on the pre-existing clinical diagnosis documented in the medical record and was not independently diagnosed or re-adjudicated using study-specific biochemical testing or diagnostic criteria. The specific diagnostic criteria applied at the time of the original clinical diagnosis were not uniformly available for retrospective verification. Individuals with documented type 1 diabetes, gestational diabetes, or secondary diabetes were excluded, and those whose diabetes type could not be established with sufficient certainty from the available clinical documentation were not eligible. Additional exclusion criteria included pregnancy, severe cognitive impairment, inability to wear either monitoring device, and acute metabolic decompensation or hospitalization during follow-up.

**Figure 1:**
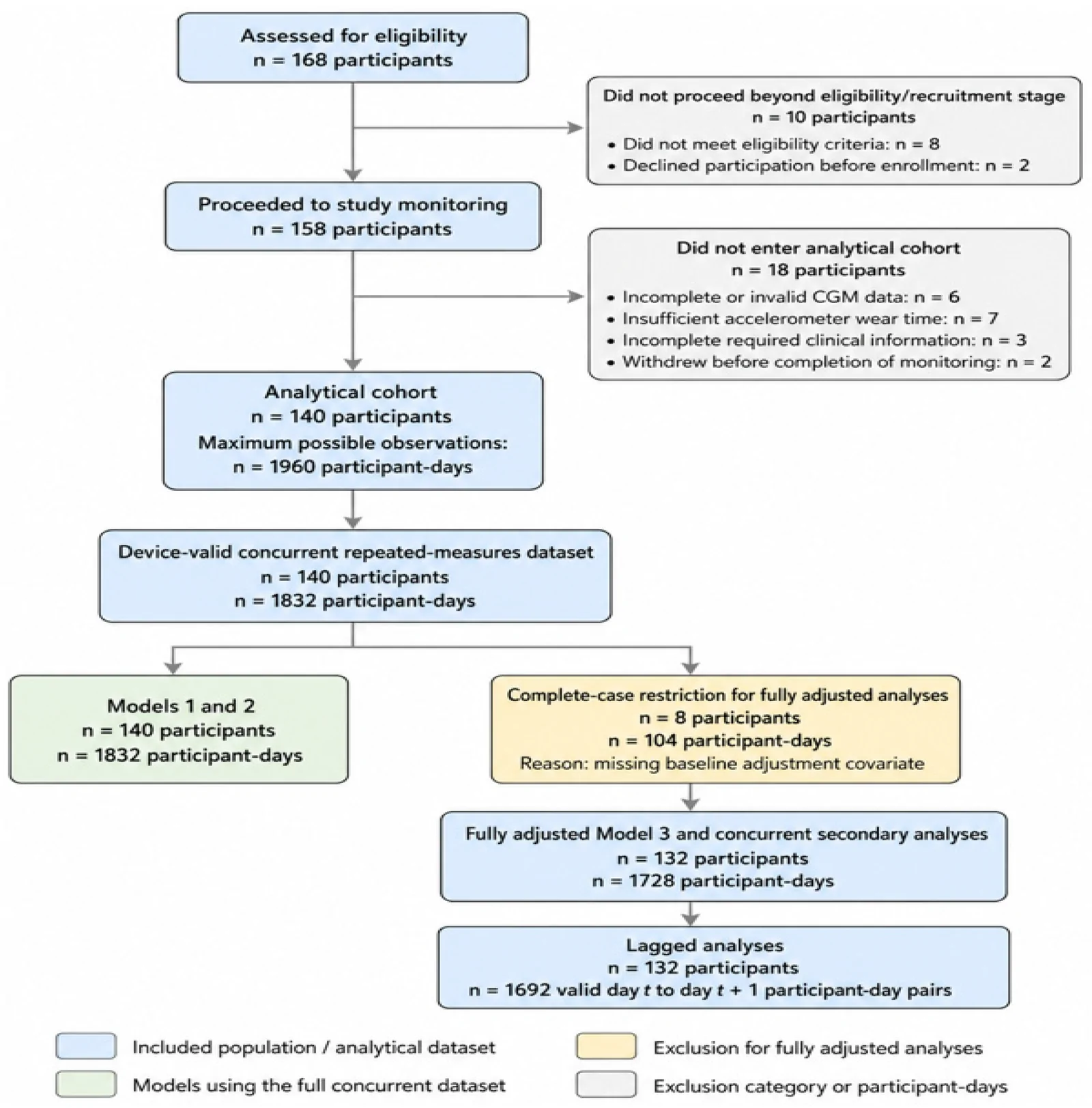
Participant and participant-day analytical flow from eligibility assessment through the concurrent and lagged repeated-measures analyses. CGM: continuous glucose monitoring.

Inclusion in the analytical cohort additionally required availability of the required clinical information and sufficient accelerometer and CGM data to satisfy the validity and temporal-overlap criteria described below. For concurrent analyses, each valid participant -day constituted one repeated observation, and participants could contribute multiple observations according to the number of days meeting these criteria. Participant and participant-day selection for the analytical sample are summarized in Figure 1.

### 2.3. Study Procedures

At baseline, sociodemographic and clinical information was collected from medical records and standardized assessments, and anthropometric measurements were performed. Participants were then fitted with the accelerometer and CGM device and received instructions for their use.

The accelerometer and CGM were used concurrently for 14 consecutive days. Participants were asked to maintain their usual diet, physical activity, medication schedule, and diabetes self-management practices and not to initiate a new structured exercise program unless medically indicated. The accelerometer was worn on the right hip during waking hours, except during bathing, swimming, or other water-based activities; accelerometer-derived movement exposures therefore represented waking wear time.

A brief monitoring diary was used to record device removal, sleep periods, unusual physical activity, illness, hypoglycemic symptoms, medication changes, and other relevant day-specific events. Diary information was used to characterize potentially atypical or confounded participant-days and to support sensitivity analyses rather than to redefine the primary accelerometer-derived exposure variables. Primary accelerometer wear and non-wear status was determined using the Choi algorithm, and diary-recorded sleep periods were not used to redefine this classification. Diary-recorded device removal was used as contextual information and did not independently determine participant-day validity. Days marked only by unusual physical activity were retained in the primary analysis because such a variation was considered part of free-living day-to-day behavior. In the diary-informed sensitivity analysis, daily sleep duration was included as a time-varying covariate, and participant-days with documented acute illness or medication changes were excluded. Detailed daily information on carbohydrate intake, meal timing, medication dose and timing, adherence, stress, and activity timing relative to meals was not available and was considered a potential source of residual time-varying confounding.

CGM sensors were applied by trained personnel or under their supervision. At the end of monitoring, accelerometer and CGM data were downloaded and evaluated for completeness, validity, and temporal overlap.

### 2.4. Accelerometer-Derived Physical Activity and Accelerometer-Defined Sedentary Time

Physical activity was assessed using ActiGraph GT3X+ accelerometers (ActiGraph LLC, Pensacola, FL, USA) worn on the right hip. Devices recorded raw acceleration at 30 Hz, and data were processed using ActiLife version 6.13.4 with the standard (Normal) filter; the Low Frequency Extension was not enabled. Vertical-axis activity counts were aggregated into 60 s epochs and expressed as counts per minute for intensity classification. This epoch length may smooth short intermittent activity bouts and was considered when interpreting intensity-specific estimates.

Non-wear time was identified using the Choi algorithm as ≥90 consecutive minutes of zero counts, allowing up to 2 min of non-zero counts when surrounded by 30 min zero-count windows [15]. A valid day required ≥10 h of wear time. For the primary analytical cohort, participants were required to contribute at least 10 valid accelerometer days, corresponding to 10 of the intended 14 monitoring days (71.4%). This prespecified participant-level completeness criterion was used to support reliable characterization of both MVPA and accelerometer-defined sedentary time and to provide a sufficiently dense monitoring profile for estimation of the participant-specific mean movement levels used in the within-person/between-person decomposition. The 10-day criterion was not a requirement of the mixed-effects model itself, which accommodated unequal numbers of valid participant-days among eligible participants. Previous reliability studies have shown that the number of monitoring days required for reliable accelerometer-derived estimates varies according to the movement metric and study population. In free-living adults monitored with a hip-worn ActiGraph GT3X+, approximately 7–10 days were required for reliable estimation of overall physical activity and MVPA, whereas 6–8 days were required for wear-time-adjusted sedentary time [16]. In older adults, a minimum of approximately 5 consecutive monitoring days was sufficient to achieve reliable estimates of physical activity and sedentary behavior [17]. On this basis, the 10-day requirement was retained as a conservative completeness criterion within the 14-day monitoring protocol. All valid participant-days with corresponding CGM data were retained; a sensitivity analysis applied a stricter requirement of ≥12 valid accelerometer days.

Accelerometer-defined sedentary time was classified as <100 counts/min, light physical activity as 100–1951 counts/min, and moderate-to-vigorous physical activity (MVPA) as ≥1952 counts/min using the Freedson adult cut-point scheme implemented in ActiLife version 6.13.4. [18]. MVPA and accelerometer-defined sedentary time were the primary movement exposures; light physical activity, steps/day, and accelerometer-defined sedentary breaks were secondary exploratory exposures. All accelerometer-derived movement variables were calculated separately for each valid participant-day. In the repeated-measures analyses, daily MVPA and accelerometer-defined sedentary time were represented using both day-specific values and participant-specific monitoring-period means: the day-specific values were expressed as deviations from each participant’s own monitoring-period mean to estimate within-person associations, whereas the participant-specific means represented between-person differences. Weekdays and weekend days were included together in the primary analyses rather than summarized as separate averages; potential differences by day type were evaluated by an additional adjustment for weekday versus weekend status in a sensitivity analysis. Because these fixed count thresholds represent absolute rather than individually calibrated intensity, classification may vary according to functional capacity or cardiorespiratory fitness. The robustness of the primary MVPA association was therefore additionally examined using a lower threshold of ≥1041 counts/min derived from an ActiGraph calibration study in older adults [19]. This alternative threshold was used as a sensitivity classification rather than as a population-specific validation threshold for adults with type 2 diabetes.

The hip-worn accelerometer does not directly identify body posture; therefore, periods < 100 counts/min were interpreted as accelerometer-defined low-movement time rather than directly measured sitting, reclining, or lying. Accelerometer-defined sedentary breaks were identified as transitions from an epoch with <100 counts/min to at least one immediately subsequent valid-wear epoch with ≥100 counts/min. Because data were aggregated into 60 s epochs, a single qualifying 1 min epoch was sufficient to constitute a break. Consecutive epochs with ≥100 counts/min following the same transition were treated as a single break; a new break was counted only after activity returned to <100 counts/min and was subsequently followed by another qualifying ≥100-counts/min epoch. Transitions spanning or immediately crossing accelerometer non-wear periods were not counted as sedentary breaks because both sides of the transition were required to consist of consecutive valid-wear epochs. Daily sedentary-break counts were calculated for each valid participant-day, and daily valid accelerometer wear time was included in the sedentary-break association model to account for differences in the opportunity to observe breaks across days. These measures therefore represent interruptions of accelerometer-defined low-movement time rather than posture-confirmed sit-to-stand transitions.

### 2.5. Continuous Glucose Monitoring

Glucose was monitored concurrently with accelerometry for 14 days using FreeStyle Libre 2 (Abbott Diabetes Care, Alameda, CA, USA). Sensor data were downloaded through LibreView web-based platform. CGM was used in an unblinded configuration, and participants could view their glucose values during monitoring.

Participant-days were considered valid when ≥70% of expected sensor readings were available. This threshold was used as a pragmatic day-level completeness criterion informed by the ≥70% data-sufficiency criterion used in international CGM reporting recommendations [8,9], while recognizing that those recommendations were developed for aggregate multi-day profiles rather than individual calendar days. Robustness to the day-level completeness threshold was evaluated by repeating the primary analysis using a stricter ≥80% CGM-completeness criterion. Daily outcomes included the mean glucose, glucose standard deviation, coefficient of variation (CV), time in range (TIR; 70–180 mg/dL), time above range (TAR; >180 mg/dL), and time below range (TBR; <70 mg/dL). A CV was calculated as the glucose standard deviation divided by the mean glucose and multiplied by 100. The GMI was not analyzed as a daily repeated-measures outcome because it is derived from the mean CGM glucose; when reported descriptively, it was calculated from the aggregated 14-day profile.

A daily CV was treated as a 24 h within-day outcome intended to characterize day-specific glucose variability rather than as a stable estimate of habitual longer-term glycemic variability. Previous CGM reliability studies have evaluated the repeatability of day-level glycemic metrics [20] and the number of monitoring days required for reliable participant-level characterization of glycemic variability [10]. Specifically, Foreman et al. reported that three monitoring days were required to achieve a reliability > 0.80 for a CV [10]. A CV calculated from a single 24 h period is therefore expected to contain greater day-specific sampling variability and measurement noise than a CV aggregated across multiple days. In a repeated-measures analysis, this day-level imprecision may increase residual variability and reduce the precision of within-person association estimates and should not be interpreted as equivalent to a stable participant-level characterization of glycemic variability. Inference in the present study was consequently based on repeated daily CV observations across the 14-day monitoring period rather than on interpreting any single-day CV as a stable participant characteristic.

Accelerometer and CGM data were synchronized by participant identifier, calendar date, and recorded clock time. Daily CGM outcomes were calculated over the full calendar day (00:00–23:59) and therefore included both daytime and overnight glucose readings, whereas accelerometer-derived movement exposures represented valid waking wear time accumulated during the corresponding calendar date. Consequently, some glucose observations, particularly those occurring overnight or in the early morning, preceded movement accumulated later that day. Same-day analyses were therefore interpreted as concurrent day-level associations rather than as temporally ordered effects. Temporal ordering was examined separately in lagged analyses relating movement on day *t* to CGM outcomes on day *t* + 1. Within-day temporally ordered windows were not evaluated.

### 2.6. Outcomes and Exposures

The primary outcome was a daily 24 h within-day CGM-derived CV. Secondary CGM outcomes were TIR, TAR, TBR, mean glucose, and glucose standard deviation. TIR, TAR, and TBR were considered components of the same daily glucose range composition because they partitioned the observed CGM time and therefore summed to approximately 100% for each participant-day. Accordingly, these three metrics were not treated as statistically independent sources of evidence; TIR characterized time within the target range, whereas TAR and TBR were interpreted as complementary components describing how time outside the target range was distributed above and below range. Analyses of these secondary outcomes were distinguished from the primary confirmatory analysis. Component-specific estimates were retained because TIR, TAR, and TBR have distinct clinical interpretations, but their associations were interpreted jointly as describing redistribution of observed CGM times across glucose ranges rather than as three independent findings.

The primary accelerometer-derived exposures were daily MVPA and accelerometer-defined sedentary time. Daily step count, light physical activity, and accelerometer-defined sedentary breaks were secondary exploratory exposures and were analyzed and reported regardless of their statistical significance. Because the opportunity to accumulate accelerometer-defined sedentary breaks depends on the duration of device observation, their exploratory association model accounted for daily valid accelerometer wear time. Valid accelerometer wear time was also incorporated into the primary movement-time modeling framework because the durations of sedentary time, light physical activity, and MVPA were constrained by daily wear time.

### 2.7. Covariates

Covariate selection followed a conceptual framework that distinguished candidate baseline confounders from markers of diabetes severity and treatment, measurement-related variables, and time-varying factors. Age, sex, diabetes duration, body mass index, hypertension, dyslipidemia, and cardiovascular disease history were treated as candidate baseline confounders for between-person adjustment because they were measured before the monitoring period and could be associated with movement behavior and glycemic outcomes. HbA1c and glucose-lowering treatment were considered clinical markers of longer-term glycemic status, diabetes severity, and treatment intensity rather than being assumed to represent pure confounders. Accordingly, the broader clinical adjustment included HbA1c together with insulin, metformin, GLP-1 receptor agonist, and SGLT2 inhibitor use. Clinical variables were obtained from medical records and standardized case-report forms, and HbA1c values were obtained when measured within 3 months of monitoring. Daily valid accelerometer wear time was treated as a measurement-related time-use variable because it determined the observation period available for accumulating accelerometer-derived movement exposures rather than representing a clinical confounder. Diary-derived sleep duration, acute illness, and medication changes were considered potential time-varying confounding factors and were evaluated in sensitivity analyses. Detailed participant-day information on dietary intake, meal timing, medication dose and timing, medication adherence, stress, and physical activity timing relative to meals was not available with sufficient resolution for statistical adjustment and was therefore considered a source of residual time-varying confounding.

### 2.8. Study Size

Study size was determined by the number of eligible participants with valid concurrent accelerometer and CGM data available during the study period. The analytical cohort comprised 140 participants, with a maximum of 1960 participant-days over 14 days; the fully adjusted complete-case primary model included 132 participants and 1728 participant-days.

No formal a priori sample-size calculation was performed for the mixed-effects model. Study precision was therefore characterized using the 95% confidence intervals of the primary within-person estimates rather than a retrospective power calculation. The confidence-interval half-width was 0.18 percentage points for the MVPA estimate and 0.04 percentage points for the accelerometer-defined sedentary time estimate. Because repeated participant-days within individuals were correlated, the participant-specific random-intercept variance, residual variance, and intraclass correlation coefficient (ICC) were also estimated and are reported with the primary results.

### 2.9. Statistical Analysis

#### 2.9.1. Descriptive Analyses

Continuous variables were summarized as means ± standard deviations or median and interquartile ranges, as appropriate, and categorical variables as frequencies and percentages. Distributional characteristics were evaluated graphically, including Q–Q plots. Correlations among movement variables and valid accelerometer wear time were examined before fitting mutually adjusted models. Baseline characteristics of participants included in the analytical cohort and those excluded because of insufficient device data were compared descriptively using absolute standardized mean differences, without null-hypothesis significance testing (Supplementary Table S1).

#### 2.9.2. Primary Analysis

The primary analysis used linear mixed-effects models with participant-specific random intercepts to examine associations of daily MVPA and accelerometer-defined sedentary time with a daily 24 h CV. Daily MVPA, accelerometer-defined sedentary time, and valid accelerometer wear time were simultaneously included and decomposed into within-person and between-person components. Within-person terms represented day-specific deviations from each participant’s monitoring-period mean, whereas between-person terms represented the corresponding participant-specific means. Because valid wear time comprised accelerometer-defined sedentary time, light physical activity, and MVPA, the within-person MVPA and sedentary time coefficients represented replacement of an equivalent duration of light physical activity within valid wear time. A supportive exploratory linear contrast estimated the association corresponding to replacement of 10 min/day of accelerometer-defined sedentary time with 10 min/day of MVPA; this estimate was not interpreted as a complete 24 h compositional analysis.

Three sequential models were fitted. Model 1 included the within-person and between-person movement and wear-time terms; Model 2 additionally adjusted for age and sex; and Model 3 further adjusted for diabetes duration, body mass index, HbA1c, insulin, metformin, GLP-1 receptor agonist and SGLT2 inhibitor use, hypertension, dyslipidemia, and cardiovascular disease history. Model 3 constituted the fully adjusted primary model. The two fully adjusted within-person associations of MVPA and accelerometer-defined sedentary time with a daily CV formed the primary confirmatory family and were adjusted using the Holm procedure.

#### 2.9.3. Secondary Analyses

Secondary linear mixed-effects models examined TIR, TAR, TBR, mean glucose, and glucose standard deviation using the same movement-time framework. Because TIR, TAR, and TBR are bounded percentages, complementary generalized models were fitted: TIR and TAR were analyzed using fractional-logit generalized estimating equations (GEE) with participant clustering, robust standard errors, and an AR(1) working correlation structure, whereas TBR was additionally evaluated using a two-part model comprising a binomial-logit GEE for any TBR and a fractional-logit GEE for TBR magnitude among participant-days with TBR > 0%. TIR, TAR, and TBR were interpreted jointly as dependent components of the daily glucose range composition. Separate component-specific models were retained to preserve the clinical interpretation of time spent within, above, and below the target range. A conventional log-ratio compositional model was not applied because TBR contained a substantial proportion of zero values, which would require an additional zero-replacement procedure before log-ratio transformation. The component-specific analyses were therefore treated as complementary rather than independent tests. A Benjamini–Hochberg false-discovery-rate correction at 5% was applied across the 10 concurrent secondary within-person exposure–outcome tests.

#### 2.9.4. Temporally Ordered Lagged Analyses

Temporally ordered analyses related MVPA and accelerometer-defined sedentary time on day *t* to CV, TIR, TAR, and TBR on day *t* + 1. Models additionally adjusted for the corresponding CGM outcome on day *t*, decomposed into within-person and between-person components, while retaining the primary movement-time and clinical adjustment structure. Participant-day pairs lacking the required exposure or outcome data were excluded. Lagged analyses were treated as exploratory and were not part of the primary confirmatory analysis. A Benjamini–Hochberg false-discovery-rate correction at 5% was applied across the eight lagged within-person tests.

#### 2.9.5. Sensitivity and Exploratory Analyses

Sensitivity analyses assessed the robustness of the primary associations using a stricter ≥12-valid-accelerometer-day criterion, ≥80% daily CGM completeness, exclusion of insulin-treated participants, additional weekday/weekend adjustment, diary-informed adjustment for daily sleep duration with exclusion of participant-days with documented acute illness or medication changes, omission of HbA1c from the fully adjusted model, an alternative MVPA threshold of ≥1041 counts/min, an AR(1) residual correlation structure, population-averaged GEE models, and single-exposure specifications. Because repeated observations were obtained on consecutive days, serial dependence was evaluated using within-participant residual autocorrelation, and the primary random-intercept model was additionally refitted with a first-order autoregressive [AR(1)] residual correlation structure to assess the robustness of the estimated associations to residual serial dependence. Model fit and residual dependence were compared using residual diagnostics, lag-1 residual autocorrelation, AIC, and BIC. The random-intercept specification was retained as the primary inferential model, while the AR(1) specification was used as a targeted robustness analysis to evaluate the sensitivity of the primary associations to residual serial dependence.

Daily step count, light physical activity, and accelerometer-defined sedentary breaks were analyzed as secondary exploratory exposures using within-person/between-person decomposition. Within-person estimates were emphasized in the main analyses, whereas corresponding between-person estimates were reported in Supplementary Table S2. Sensitivity and exploratory analyses were not treated as additional confirmatory hypothesis families.

Missing baseline covariates were handled using complete-case analysis when variable-specific missingness was <5%. Model diagnostics included residual-versus-fitted and Q–Q plots and assessment of within-participant residual autocorrelation; distributional and model-specific diagnostics were additionally examined for the bounded CGM outcomes and generalized models. For the linear mixed-effects models of TIR, TAR, and TBR, fitted values were additionally inspected for predictions outside the admissible 0–100% range. All tests were two-sided, and the effect estimates are reported with 95% confidence intervals and nominal or multiplicity-adjusted *p*- or *q*-values, as applicable.

### 2.10. Software and Code Availability

Accelerometer data were processed using ActiLife version 6.13.4, and CGM data were downloaded through LibreView. Data management and statistical analyses were performed using Microsoft Excel for Microsoft 365 and R version 4.4.2 (R Foundation for Statistical Computing, Vienna, Austria), including the lme4 version 1.1-35.5 [21], lmerTest version 3.1-3 [22], geepack version 1.3.12 [23], tidyverse version 2.0.0, performance, and nlme version 3.1-166 packages.

Analytical code for data cleaning, accelerometer–CGM synchronization, variable derivation, and statistical analysis is available from the corresponding author upon reasonable request. The code is not publicly posted because it contains institutional data-processing paths and variable structures linked to the restricted clinical dataset; it contains no personally identifiable participant information.

### 2.11. Ethics

This study was conducted in accordance with the Declaration of Helsinki and was approved by the Institutional Research Ethics Committee of Universidad Nacional de Educación Enrique Guzmán y Valle (UNE-REC-2024-013; 25 November 2024), with authorization from the participating clinical site in Lima, Peru. Written informed consent was obtained before any study procedures were initiated. Participant confidentiality was protected through coded analytical data, removal of direct identifiers, and storage on password-protected institutional servers accessible only to authorized study personnel.

## 3. Results

### 3.1. Analytical Sample and Valid Monitoring Data

A total of 168 adults were assessed for eligibility. Eight did not meet the eligibility criteria, leaving 160 eligible individuals. Two otherwise eligible individuals declined participation before enrollment, and 158 proceeded to study monitoring. Of these, 18 did not enter the analytical cohort because of incomplete or invalid CGM data (*n* = 6), insufficient accelerometer wear time (*n* = 7), incomplete required clinical information (*n* = 3), or withdrawal before completion of monitoring (*n* = 2). The resulting analytical cohort comprised 140 participants (Figure 1).

Across the 14-day monitoring period, these 140 participants could contribute a maximum of 1960 participant-days. After application of the accelerometer, CGM, and temporal-overlap validity criteria, 1832 participant-days (93.5%) were available for concurrent repeated-measures analyses. Participants contributed a median of 13 valid accelerometer days (IQR, 12–14) and 14 valid CGM days (IQR, 13–14), with a mean accelerometer wear time of 13.8 ± 1.7 h/day and mean CGM data availability of 87.2 ± 7.8%.

Baseline characteristics of participants included in the analytical cohort and those excluded because of insufficient device data are presented in Supplementary Table S1. No substantial imbalances were observed across the measured baseline characteristics in this descriptive comparison (all absolute SMDs < 0.20), although small differences were present for age, body mass index, HbA1c, insulin use, and hypertension.

Models 1 and 2 used the 140-participant device-valid dataset. Complete-case restrictions for baseline adjustment covariates resulted in 132 participants contributing 1728 participant-days to the fully adjusted analyses; details of covariate missingness are reported in Section 3.9.

### 3.2. Baseline Characteristics

Baseline characteristics are presented in Table 1. The 140 participants had a mean age of 59.8 ± 9.7 years, 55.7% were men, and the median duration of type 2 diabetes was 9.0 years (IQR, 5.0–14.0). The mean HbA1c was 7.8 ± 1.2%.

### 3.3. Accelerometer-Derived Movement Metrics

Table 2 summarizes accelerometer-derived movement characteristics across valid participant-days, including MVPA, accelerometer-defined sedentary time, light physical activity, daily steps, and accelerometer-defined sedentary breaks. The mean MVPA was 32.6 ± 18.9 min/day, and the mean accelerometer-defined sedentary time was 532.4 ± 96.8 min/day.

### 3.4. Continuous Glucose Monitoring-Derived Metrics

Table 3 summarizes the CGM characteristics and daily CGM-derived metrics. Monitoring completeness and continuous glucose metrics are presented in Table 3, Panel A, whereas the distribution of the bounded glucose range metrics is summarized in Table 3, Panel B. The mean daily 24 h CV was 35.8 ± 8.1%, TIR was 62.4 ± 18.9%, TAR was 35.1 ± 18.7%, and TBR was 2.5 ± 3.5%. TBR showed a pronounced lower-bound concentration, with 54.8% of participant-days having TBR = 0%.

### 3.5. Primary Repeated-Measures Analysis

Primary mutually adjusted movement-time associations are presented in Table 4. The fully adjusted analysis included 132 participants contributing 1728 participant-days. On days when MVPA was 10 min/day higher than a participant’s own monitoring-period average, a daily 24 h CV was, on average, 0.27 percentage points lower (95% CI: −0.45 to −0.09; nominal *p* = 0.003; Holm-adjusted *p* = 0.006). On days when accelerometer-defined sedentary time was 10 min/day higher than a participant’s own monitoring-period average, a daily CV was, on average, 0.05 percentage points higher (95% CI: 0.01 to 0.09; nominal *p* = 0.014; Holm-adjusted *p* = 0.014).

Replacing 10 min/day of accelerometer-defined sedentary time with 10 min/day of MVPA within valid accelerometer wear time was associated with a 0.32-percentage-point lower CV (β = −0.32, 95% CI: −0.50 to −0.14; *p* < 0.001). Between-person estimates were directionally consistent with the within-person associations; the corresponding coefficients and 95% confidence intervals are provided in Table 4. In the fully adjusted model, the participant-specific random-intercept variance was 18.4, and the residual variance was 47.2, corresponding to an ICC of 0.280.

### 3.6. Secondary Repeated-Measures Analyses

Concurrent secondary-outcome associations are summarized in Table 5. On days when MVPA was 10 min/day higher than a participant’s own monitoring-period average, TIR was, on average, 0.82 percentage points higher (95% CI: 0.38 to 1.26) and TAR was 0.78 percentage points lower (95% CI: −1.21 to −0.35). Conversely, on days when accelerometer-defined sedentary time was 10 min/day higher than a participant’s own monitoring-period average, TIR was, on average, 0.19 percentage points lower (95% CI: −0.32 to −0.06) and TAR was 0.18 percentage points higher (95% CI: 0.05 to 0.30). Higher within-person MVPA was also concurrently associated with lower mean glucose and glucose standard deviations, whereas higher within-person accelerometer-defined sedentary time showed associations in the opposite direction; these associations remained statistically supported after Benjamini–Hochberg correction (all FDR *q* ≤ 0.018). Neither movement exposure showed a statistically detectable association with TBR. Because TIR, TAR, and TBR partition the same daily CGM observation time, these component-specific estimates were interpreted jointly and not as independent corroborating associations. Fitted values from the linear mixed-effects models for TIR, TAR, and TBR remained within the admissible 0–100% range. Complementary generalized analyses specifically accounting for the bounded distributions of these outcomes yielded the same overall interpretation (Table 5, Panel B). Corresponding between-person estimates are reported in Supplementary Table S2.

### 3.7. Lagged Analyses

Lagged analyses included 132 participants contributing 1692 valid day-*t* to day-*t* + 1 participant-day pairs. Associations of MVPA and accelerometer-defined sedentary time with a next-day CV, TIR, and TAR were generally directionally consistent with the concurrent findings; however, none of the eight lagged tests remained statistically significant after Benjamini–Hochberg correction (minimum FDR *q* = 0.061). Neither movement exposure showed a statistically detectable association with next-day TBR. Complementary generalized analyses yielded directionally consistent estimates. Full lagged estimates are provided in Supplementary Table S3.

### 3.8. Sensitivity and Exploratory Secondary-Exposure Analyses

The primary within-person associations of higher MVPA with a lower daily CV and greater accelerometer-defined sedentary time with a higher daily CV were materially unchanged across sensitivity analyses addressing a stricter ≥ 80% daily CGM-completeness threshold, accelerometer-data completeness, insulin treatment, weekday/weekend status, diary-informed time-varying factors, HbA1c adjustment, MVPA intensity classification, residual serial correlation, model specification, and single-exposure models. The primary random-intercept model showed modest residual lag-1 autocorrelation (0.17); incorporating an AR(1) residual structure reduced this to 0.02 (ρ = 0.16) and improved model fit (AIC, 11,828.90 vs. 11,846.20; BIC, 11,951.40 vs. 11,962.50), while yielding materially unchanged estimates for MVPA (β = −0.26 vs. −0.27) and accelerometer-defined sedentary time (β = 0.05 in both specifications). Under the AR(1) specification, the within-person MVPA association was β = −0.26 percentage points per 10 min/day (95% CI, −0.44 to −0.08; nominal *p* = 0.005), and the accelerometer-defined sedentary time association was β = 0.05 percentage points per 10 min/day (95% CI, 0.01 to 0.09; nominal *p* = 0.017). Thus, both the magnitude and inferential interpretation of the primary associations were materially unchanged after explicit modeling of residual serial correlation.

Exploratory analyses showed an inverse within-person association between daily step count and CV (β = −0.18 per 1000 steps/day, 95% CI: −0.31 to −0.05; *p* = 0.007), whereas associations for light physical activity and accelerometer-defined sedentary breaks were statistically inconclusive. Corresponding between-person estimates for daily step count, light physical activity, and accelerometer-defined sedentary breaks are reported in Supplementary Table S2. Full sensitivity, serial-correlation, and exploratory secondary-exposure results are provided in Supplementary Table S4.

### 3.9. Missing Data

After application of the prespecified device-validity criteria, no missing data remained for the primary movement exposures or daily CGM-derived CV. Eight participants (5.7%) had missing data for at least one baseline covariate required for the fully adjusted models. Because variable-specific missingness was <5%, complete-case analysis was used. These participants contributed 104 of the 1832 otherwise device-valid participant-days, resulting in 132 participants and 1728 participant-days for the fully adjusted analyses. Participants with missing adjustment covariates remained eligible for analyses that did not require those variables.

## 4. Discussion

### 4.1. Principal Findings

In this longitudinal repeated-measures study, on days when MVPA was higher than a participant’s own monitoring-period average, within-day glycemic variability was lower, whereas greater-than-usual accelerometer-defined sedentary time was associated with higher glycemic variability. The secondary concurrent findings were directionally consistent with these primary associations, although the observed associations were modest and should not be interpreted causally. A principal contribution of this study is the evaluation of day-level movement–glycemia associations while explicitly separating within-person variations from between-person differences, complementing previous free-living studies that used monitoring-period summaries, same-day comparisons, or cross-sectional association analyses [6,11,12,13,14].

### 4.2. Physical Activity and Glycemic Outcomes

The inverse association between within-person MVPA and a daily CV is broadly consistent with experimental evidence that exercise can favorably influence CGM-derived glucose profiles in adults with type 2 diabetes. Meta-analyses of CGM studies have reported reductions in mean 24 h glucose, hyperglycemic exposure, and indices of glycemic variability following short-term or acute postprandial exercise [24,25]. Free-living evidence is more limited; El Fatouhi et al. reported associations between device-measured daily step counts and subsequent glycemic indices, although their study involved adults without diabetes and evaluated different exposure–outcome relationships [14]. These findings support the biological plausibility of the present associations while emphasizing that naturally occurring free-living MVPA is not equivalent to experimentally prescribed exercise.

Skeletal muscle contraction can increase glucose uptake through insulin-independent pathways during activity, and exercise may enhance insulin sensitivity afterward [3]. These mechanisms provide biological plausibility for the observed association between movement and glucose patterns, but they were not directly assessed in the present study and do not establish the temporal direction of the observed relationship. Meal composition and timing, medication dose and timing, and the temporal relationship between physical activity and meals were not measured with sufficient resolution to identify specific pathways. The magnitude of the observed associations was modest, and their clinical significance remains uncertain. Because the present study was observational and evaluated naturally occurring day-to-day movement rather than prescribed exercise, these associations should not be interpreted as defining a specific exercise dose or as demonstrating that a particular movement-time reallocation would produce a corresponding glycemic benefit. The model-based reallocation contrast should likewise be interpreted as an association within observed accelerometer wear time rather than as a clinical prescription. Whether associations of this magnitude translate into clinically meaningful glycemic benefits when sustained over longer periods requires prospective intervention studies.

### 4.3. Accelerometer-Defined Sedentary Time and Glycemic Outcomes

Greater accelerometer-defined sedentary time was associated with higher glycemic variability and a less favorable concurrent pattern of TIR and TAR. Because sedentary time was derived from a hip-worn accelerometer using a <100 counts/min threshold, it represents accelerometer-defined low-movement time rather than posture-confirmed sitting or reclining. Within the constrained movement-time model, the sedentary time coefficient represented replacement of light physical activity with accelerometer-defined sedentary time, while the direct sedentary-time-to-MVPA contrast represented a model-based reallocation within observed wear time rather than a complete 24 h compositional substitution.

The direction of these findings is broadly consistent with previous work in adults with type 2 diabetes. Paing et al. reported that a greater sedentary time was associated with less time in euglycemia and that more frequent sedentary breaks were associated with more time in euglycemia [6]. In a randomized crossover study, Duvivier et al. found that replacing prolonged sitting with standing and light-intensity walking reduced 24 h glucose exposure compared with a sitting regimen [7]. Direct comparison remains limited because these studies differed in movement assessment, exposure definitions, intervention conditions, and glycemic outcomes.

### 4.4. Secondary, Exploratory, and Temporally Ordered Findings

The concordant direction of the secondary concurrent outcomes suggests that the day-level associations of movement behavior extended beyond glycemic variability to the broader same-day CGM profile. This pattern is consistent with previous free-living evidence in adults with type 2 diabetes. Gauthier et al. reported lower same-day glycemic variability on days with greater physical activity [11], while studies by Lee et al. linked greater daytime step counts and lower sedentary time with more favorable CGM profiles [12,13]. However, differences in movement assessment, treatment characteristics, outcome definitions, and analytical approaches limit direct comparison across studies.

In contrast, the temporally ordered analyses did not provide statistically supported evidence of next-day associations after multiplicity correction. This temporal pattern differs from that reported by El Fatouhi et al., who found associations between higher daily step counts and selected next-day glycemic indices, although their study involved adults without diabetes and evaluated a different movement exposure [14]. Accordingly, the present lagged findings should be regarded as exploratory and do not establish a next-day carryover effect or causal temporal relationship.

The absence of a statistically detectable association with TBR should likewise not be interpreted as evidence of absence of hypoglycemia risk, given the concentration of participant-days with no below-range exposure and the lack of event-level information on activity and treatment timing. The exploratory inverse association between daily step count and a CV is directionally consistent with previous wearable-based findings [12,13], whereas the inconclusive estimates for light physical activity and accelerometer-defined sedentary breaks do not support specific behavioral targets.

### 4.5. Practical and Clinical Implications

The present findings indicate that day-to-day movement behavior is associated with modest differences in concurrent CGM-derived glucose profiles. This observational pattern is broadly consistent with previous free-living studies linking device-measured physical activity or sedentary behavior with CGM-derived glycemic outcomes, although study populations, movement measures, and analytical approaches have differed [6,11,12,13,14]. These findings therefore support consideration of movement behavior as one component of the behavioral context in which daily CGM patterns occur, but they do not establish that modifying movement behavior would produce a corresponding improvement in glycemic outcomes.

Joint assessment of objectively measured movement and CGM could be evaluated in future studies as a strategy for contextualizing individual glucose patterns. However, the present study did not evaluate a counseling intervention, clinical decision-support strategy, or patient outcomes resulting from joint review of accelerometer and CGM data. Accordingly, the findings do not support incorporating paired monitoring into routine clinical care, prescribing a specific exercise dose, or recommending a particular movement-time target. Prospective intervention studies are needed to determine whether such applications provide clinically meaningful benefit.

### 4.6. Strengths and Limitations

Strengths of this study include the objective and concurrent assessment of movement behavior and glucose over repeated participant-days under free-living conditions, the separation of within-person from between-person associations, and the use of multiplicity adjustment and sensitivity analyses to assess the robustness of the primary findings.

Several limitations should be considered. First, the observational design precludes causal inference. Same-day accelerometer exposures represented waking wear time whereas CGM outcomes covered the full 24 h calendar day, and the temporally ordered analyses, although informative about sequence, remained observational. Second, within-person decomposition reduces the influence of stable between-person characteristics but does not control for time-varying factors such as diet, medication dose and timing, sleep, stress, or activity timing relative to meals, which were not measured with sufficient day-level resolution. Third, both accelerometry and CGM are subject to measurement limitations, and the isotemporal contrasts represent reallocations within observed accelerometer wear time rather than a complete 24 h movement composition. Complete-case adjustment may also have introduced selection bias, and generalizability is limited by consecutive non-probability recruitment from a tertiary outpatient service in Lima, Peru, and the requirement for successful dual-device monitoring.

Future studies should use prospective or experimental designs with longer monitoring and more temporally resolved assessment of movement, meals, medication, sleep, and other day-level factors. Multidimensional monitoring approaches may facilitate this type of investigation [26].

## 5. Conclusions

In this longitudinal repeated-measures study of adults with type 2 diabetes, higher daily MVPA was associated with lower 24 h within-day glycemic variability and a more favorable concurrent CGM profile, whereas greater accelerometer-defined sedentary time showed the opposite pattern. These associations represented modest day-to-day differences in CGM-derived glycemic metrics, and the clinical significance of their magnitude remains to be established. The temporally ordered estimates were generally directionally consistent with the concurrent findings but did not remain statistically supported after correction for multiple testing and should therefore be considered exploratory. No statistically detectable association with TBR was observed for either movement exposure; however, this finding should not be interpreted as evidence of absence of hypoglycemia risk.

The findings support further investigation of objectively measured day-level movement patterns as potentially modifiable correlates of glycemic variability in adults with type 2 diabetes. Because the study was observational, the results do not establish causality or a specific exercise dose or movement-time reallocation for clinical practice. Longer longitudinal studies and controlled interventions incorporating temporally resolved information on movement, meals, medication use, and CGM outcomes are needed to determine whether sustained changes in the amount, timing, or composition of movement produce clinically meaningful glycemic benefits while adequately characterizing one’s hypoglycemia risk.

## Figures and Tables

**Table 1:** Baseline characteristics of the study participants.

Variable	Total Sample (*n* = 140)
Age, years	59.8 ± 9.7
Male sex, *n* (%)	78 (55.7)
Diabetes duration, years	9.0 (IQR, 5.0–14.0)
Body mass index, kg/m^2^	29.6 ± 4.8
HbA1c, %	7.8 ± 1.2
Insulin use, *n* (%)	52 (37.1)
Metformin use, *n* (%)	112 (80.0)
GLP-1 receptor agonist use, *n* (%)	21 (15.0)
SGLT2 inhibitor use, *n* (%)	34 (24.3)
Hypertension, *n* (%)	86 (61.4)
Dyslipidemia, *n* (%)	92 (65.7)
Cardiovascular disease history, *n* (%)	26 (18.6)

Note: Values are presented as means ± standard deviations, medians (IQRs), or *n* (%), as appropriate. IQR: interquartile range; HbA1c: glycated hemoglobin; GLP-1: glucagon-like peptide-1; SGLT2: sodium–glucose cotransporter 2.

**Table 2:** Accelerometer-derived physical activity and accelerometer-defined sedentary time metrics.

Accelerometer-Derived Variable	Daily Participant-Day Values
Valid accelerometer days per participant	13 (IQR, 12–14)
Accelerometer wear time, h/day	13.8 ± 1.7
MVPA, min/day	32.6 ± 18.9
Light physical activity, min/day	245.7 ± 62.5
Accelerometer-defined sedentary time, min/day	532.4 ± 96.8
Steps/day	7420 ± 2860
Accelerometer-defined sedentary breaks/day	42.3 ± 12.1

Note: Values are presented as means ± standard deviations or medians (IQRs), as appropriate. Participant-day values were calculated using valid monitoring days with concurrent accelerometer and continuous glucose monitoring data. Accelerometer-defined sedentary breaks were defined as transitions from <100 counts/min to ≥100 counts/min lasting at least 1 min and therefore represent changes in accelerometer-defined movement intensity rather than posture-confirmed sit-to-stand transitions. IQR: interquartile range; MVPA: moderate-to-vigorous physical activity.

**Table 3:** Continuous glucose monitoring-derived metrics.

**Panel A.** CGM Characteristics and Continuous Glucose Metrics			
**CGM-Derived Metric**		**Daily Participant-Day Values**	
Valid CGM days per participant		14 (IQR, 13–14)	
CGM data availability, %		87.2 ± 7.8	
Mean glucose, mg/dL		163.7 ± 29.2	
Glucose standard deviation, mg/dL		58.3 ± 18.1	
24 h coefficient of variation, %		35.8 ± 8.1	
**Panel B.** Distribution of daily CGM glucose range metrics			
**CGM-derived metric**	**Mean ± SD, %**	**Median (IQR), %**	**Observed range, %**
Time in range	62.4 ± 18.9	63.1 (49.4–76.5)	4.8–99.4
Time above range	35.1 ± 18.7	33.5 (21.1–47.7)	0.4–93.1
Time below range	2.5 ± 3.5	0.0 (0.0–4.8)	0.0–21.6

Note: TIR, TAR, and TBR partition valid CGM observation time and sum to 100% apart from rounding. TIR was defined as 70–180 mg/dL, TAR as >180 mg/dL, and TBR as <70 mg/dL; 54.8% of participant-days had TBR = 0%. A CV represents 24 h within-day glycemic variability calculated for each valid participant-day. CGM: continuous glucose monitoring; CV: coefficient of variation; IQR: interquartile range; SD: standard deviation; TAR: time above range; TBR: time below range; TIR: time in range.

**Table 4:** Primary mutually adjusted movement-time associations and isotemporal substitution estimates for a daily 24 h CGM-derived coefficient of variation.

**Panel A.** Mutually Adjusted Repeated-Measures Movement-Time Model						
**Exposure**	**Association Component**	**Model**	**β Coefficient**	**95% CI**	**Nominal *p*-Value**	**Holm-Adjusted *p*-Value**
MVPA, per 10 min/day	Within-person	Model 1	−0.38	−0.55 to −0.21	<0.001	—
MVPA, per 10 min/day	Within-person	Model 2	−0.32	−0.50 to −0.14	<0.001	—
MVPA, per 10 min/day	Within-person	Model 3, fully adjusted	−0.27	−0.45 to −0.09	0.003	0.006
MVPA, per 10 min/day	Between-person	Model 3, fully adjusted	−0.36	−0.68 to −0.04	0.028	—
Accelerometer-defined sedentary time, per 10 min/day	Within-person	Model 1	0.08	0.04 to 0.12	<0.001	—
Accelerometer-defined sedentary time, per 10 min/day	Within-person	Model 2	0.07	0.03 to 0.11	0.001	—
Accelerometer-defined sedentary time, per 10 min/day	Within-person	Model 3, fully adjusted	0.05	0.01 to 0.09	0.014	0.014
Accelerometer-defined sedentary time, per 10 min/day	Between-person	Model 3, fully adjusted	0.08	0.02 to 0.14	0.009	—
**Panel B.** Within-person isotemporal substitution contrasts from the fully adjusted model						
**Time substitution, per 10 min/day**		**β coefficient**	**95% CI**	***p*-value**		
Light physical activity → MVPA		−0.27	−0.45 to −0.09	0.003		
Light physical activity → accelerometer-defined sedentary time		0.05	0.01 to 0.09	0.014		
Accelerometer-defined sedentary time → MVPA		−0.32	−0.50 to −0.14	<0.001		

Note: β coefficients represent percentage-point differences in daily 24 h CGM-derived CV. Models 1 and 2 included 140 participants contributing 1832 participant-days; Model 3 included 132 participants contributing 1728 participant-days. All models included within-person and between-person components of MVPA, accelerometer-defined sedentary time, and valid accelerometer wear time. Model 2 additionally adjusted for age and sex, and Model 3 used the full covariate set described in Section 2.9. With valid wear time included, light physical activity was the omitted movement component. Holm adjustment was applied to the two fully adjusted within-person primary associations; indicates not applicable. Panel B reports within-person movement-time reallocation contrasts. CI: confidence interval; CGM: continuous glucose monitoring; CV: coefficient of variation; MVPA: moderate-to-vigorous physical activity.

**Table 5:** Fully adjusted repeated-measures associations with secondary CGM-derived outcomes.

**Panel A.** Linear Mixed-Effects Models							
**Outcome**	**Exposure**	**Association Component**	**β Coefficient**	**95% CI**	**Nominal *p*-Value**	**FDR *q*-Value**	
Time in range, %	MVPA, per 10 min/day	Within-person	0.82	0.38 to 1.26	<0.001	<0.005	
Time above range, %	MVPA, per 10 min/day	Within-person	−0.78	−1.21 to −0.35	<0.001	<0.005	
Time below range, %	MVPA, per 10 min/day	Within-person	−0.04	−0.12 to 0.04	0.327	0.361	
Mean glucose, mg/dL	MVPA, per 10 min/day	Within-person	−1.45	−2.38 to −0.52	0.002	0.006	
Glucose SD, mg/dL	MVPA, per 10 min/day	Within-person	−0.71	−1.17 to −0.25	0.003	0.006	
Time in range, %	Accelerometer-defined sedentary time, per 10 min/day	Within-person	−0.19	−0.32 to −0.06	0.004	0.007	
Time above range, %	Accelerometer-defined sedentary time, per 10 min/day	Within-person	0.18	0.05 to 0.30	0.006	0.009	
Time below range, %	Accelerometer-defined sedentary time, per 10 min/day	Within-person	0.01	−0.01 to 0.04	0.361	0.361	
Mean glucose, mg/dL	Accelerometer-defined sedentary time, per 10 min/day	Within-person	0.35	0.12 to 0.58	0.003	0.006	
Glucose SD, mg/dL	Accelerometer-defined sedentary time, per 10 min/day	Within-person	0.16	0.03 to 0.29	0.014	0.018	
**Panel B.** Generalized sensitivity analyses for bounded CGM-derived percentage outcomes							
**Outcome**	**Exposure**	**Model**	**Effect measure**		**Estimate**	**95% CI**	***p*** **-value**
Time in range	MVPA, per 10 min/day	Fractional-logit GEE	Average marginal effect, percentage points		0.79	0.36 to 1.22	<0.001
Time above range	MVPA, per 10 min/day	Fractional-logit GEE	Average marginal effect, percentage points		−0.75	−1.17 to −0.33	<0.001
Time in range	Accelerometer-defined sedentary time, per 10 min/day	Fractional-logit GEE	Average marginal effect, percentage points		−0.18	−0.31 to −0.05	0.006
Time above range	Accelerometer-defined sedentary time, per 10 min/day	Fractional-logit GEE	Average marginal effect, percentage points		0.17	0.05 to 0.29	0.007
Any TBR (>0% vs. 0%)	MVPA, per 10 min/day	Binomial-logit GEE	Odds ratio		0.97	0.92 to 1.03	0.318
TBR magnitude among TBR >0% days	MVPA, per 10 min/day	Fractional-logit GEE	Average marginal effect, percentage points		−0.03	−0.09 to 0.03	0.329
Any TBR (>0% vs. 0%)	Accelerometer-defined sedentary time, per 10 min/day	Binomial-logit GEE	Odds ratio		1.01	0.99 to 1.03	0.358
TBR magnitude among TBR >0% days	Accelerometer-defined sedentary time, per 10 min/day	Fractional-logit GEE	Average marginal effect, percentage points		0.01	−0.01 to 0.04	0.382

Note: The fully adjusted concurrent secondary-outcome analyses included 132 participants contributing 1728 participant-days. Panel A reports within-person associations per 10 min/day using linear mixed-effects models with the Model 3 covariate set described in Section 2.9; Benjamini–Hochberg FDR correction was applied across the 10 concurrent secondary exposure–outcome tests. Panel B presents complementary generalized analyses for bounded outcomes. TIR and TAR were analyzed using fractional-logit GEE, and TBR was analyzed using a two-part model because 54.8% of participant-days had TBR = 0%. Average marginal effects are expressed in percentage points. CI: confidence interval; CGM: continuous glucose monitoring; FDR: false discovery rate; GEE: generalized estimating equations; MVPA: moderate-to-vigorous physical activity; SD: standard deviation; TAR: time above range; TBR: time below range; TIR: time in range.

## Data Availability

The data that support the findings of this study are available from the corresponding author upon reasonable request. The dataset contains information that could compromise the privacy of study participants and is therefore not publicly shared. Access will be granted to qualified researchers who present an ethically approved protocol and agree to a data-use agreement.

## Supplementary Materials

The following supporting information can be downloaded at: https://www.mdpi.com/article/10.3390/jfmk11030338/s1, Table S1: Baseline characteristics of participants included in the analytical cohort and those excluded because of insufficient accelerometer or CGM data; Table S2: Between-person associations from secondary concurrent, lagged, and exploratory repeated-measures analyses; Table S3: Lagged associations between day-*t* movement behaviors and day-*t* + 1 CGM-derived outcomes, adjusted for the corresponding day-*t* glycemic outcome; Table S4: Sensitivity and exploratory analyses for the primary associations with daily 24 h CGM-derived coefficient of variations.
