# Supplementary material for: Accelerometer-Derived Physical Activity, Sedentary Behavior, and Continuous Glucose Monitoring-Derived Glycemic Variability in Adults with Type 2 Diabetes: A Longitudinal Repeated-Measures Study
Source: J Funct Morphol Kinesiol. 2026 Aug 28;11(3):338. doi: 10.3390/jfmk11030338 (PMC13608073; doi:10.3390/jfmk11030338)
Supplement: Supplementary file 1 [file jfmk-11-00338-s001.zip › Supplementary Table S1. Baseline characteristics.pdf]

Supplementary Table S1. Baseline characteristics of participants included in the analytical cohort and those excluded because of insufficient accelerometer or continuous glucose monitoring data.

Variable	Analytical cohort (n = 140)	Excluded for insufficient device data (n = 13)	Absolute SMD
Age, years	59.8 ± 9.7	61.2 ± 10.3	0.14
Male sex, n (%)	78 (55.7)	7 (53.8)	0.04
Diabetes duration, years	9.0 (IQR, 5.0–14.0)	10.0 (IQR, 6.0–15.0)	0.14
Body mass index, kg/m^2	29.6 ± 4.8	30.4 ± 5.1	0.17
HbA1c, %	7.8 ± 1.2	8.0 ± 1.3	0.17
Insulin use, n (%)	52 (37.1)	6 (46.2)	0.18
Metformin use, n (%)	112 (80.0)	10 (76.9)	0.07
GLP-1 receptor agonist use, n (%)	21 (15.0)	2 (15.4)	0.01
SGLT2 inhibitor use, n (%)	34 (24.3)	3 (23.1)	0.03
Hypertension, n (%)	86 (61.4)	9 (69.2)	0.16
Dyslipidemia, n (%)	92 (65.7)	9 (69.2)	0.08
Cardiovascular disease history, n (%)	26 (18.6)	3 (23.1)	0.11

Note: Values are presented as mean ± standard deviation, median (interquartile range), or n (%), as appropriate. Absolute standardized mean differences (SMDs) were used to characterize between-group imbalance. No null-hypothesis significance testing was performed for this descriptive comparison. IQR: interquartile range; HbA1c: glycated hemoglobin; GLP-1: glucagon-like peptide-1; SGLT2: sodium–glucose cotransporter 2.
