# Supplementary material for: Accelerometer-Derived Physical Activity, Sedentary Behavior, and Continuous Glucose Monitoring-Derived Glycemic Variability in Adults with Type 2 Diabetes: A Longitudinal Repeated-Measures Study
Source: J Funct Morphol Kinesiol. 2026 Aug 28;11(3):338. doi: 10.3390/jfmk11030338 (PMC13608073; doi:10.3390/jfmk11030338)
Supplement: Supplementary file 1 [file jfmk-11-00338-s001.zip › Supplementary Table S2. Between-person associations from secondary concurrent.pdf]

Supplementary Table S2. Between-person associations from secondary concurrent, lagged, and exploratory repeated-measures analyses.

Panel A. Between-person associations of primary movement exposures with concurrent secondary CGM outcomes

Exposure	Outcome	Exposure increment	β coefficient	95% CI	Nominal p value
MVPA	Time in range, %	10 min/day	1.05	0.15 to 1.95	0.023
MVPA	Time above range, %	10 min/day	−0.98	−1.86 to −0.10	0.029
MVPA	Time below range, %	10 min/day	−0.07	−0.23 to 0.09	0.39
MVPA	Mean glucose, mg/dL	10 min/day	−1.90	−3.65 to −0.15	0.034
MVPA	Glucose SD, mg/dL	10 min/day	−0.92	−1.78 to −0.06	0.036
Accelerometer-defined sedentary time	Time in range, %	10 min/day	−0.29	−0.55 to −0.03	0.029
Accelerometer-defined sedentary time	Time above range, %	10 min/day	0.27	0.02 to 0.52	0.035
Accelerometer-defined sedentary time	Time below range, %	10 min/day	0.02	−0.03 to 0.07	0.43
Accelerometer-defined sedentary time	Mean glucose, mg/dL	10 min/day	0.52	0.04 to 1.00	0.034
Accelerometer-defined sedentary time	Glucose SD, mg/dL	10 min/day	0.25	0.01 to 0.49	0.041

Panel B. Between-person movement estimates from lagged models

Exposure on day *t*	Outcome on day *t* + 1	Exposure increment	β coefficient	95% CI	Nominal p value
MVPA	Coefficient of variation, %	10 min/day	−0.14	−0.45 to 0.17	0.376
MVPA	Time in range, %	10 min/day	0.28	−0.60 to 1.16	0.532
MVPA	Time above range, %	10 min/day	−0.25	−1.11 to 0.61	0.568
MVPA	Time below range, %	10 min/day	−0.03	−0.18 to 0.12	0.694
Accelerometer-defined sedentary time	Coefficient of variation, %	10 min/day	0.03	−0.03 to 0.09	0.327
Accelerometer-defined sedentary time	Time in range, %	10 min/day	−0.06	−0.31 to 0.19	0.638
Accelerometer-defined sedentary time	Time above range, %	10 min/day	0.05	−0.19 to 0.29	0.683
Accelerometer-defined sedentary time	Time below range, %	10 min/day	0.01	−0.04 to 0.06	0.695

Panel C. Between-person associations of secondary exploratory movement exposures with daily 24-h CV

Secondary exploratory exposure	Exposure increment	β coefficient	95% CI	Nominal p value
Daily step count	1000 steps/day	−0.24	−0.47 to −0.01	0.041
Light physical activity	30 min/day	−0.12	−0.28 to 0.04	0.142
Accelerometer-defined sedentary breaks	10 breaks/day	−0.17	−0.36 to 0.02	0.079

Note: Between-person terms represent cross-participant differences associated with participants' average exposure levels over the monitoring period and should not be interpreted as day-to-day within-person effects. Panels A and C included 132 participants contributing 1728 participant-days; Panel B included 132 participants contributing 1692 valid day-*t* to day-*t* + 1 participant-day pairs. All models used the fully adjusted Model 3 clinical covariate set described in Section 2.9.2. Panel A presents between-person estimates from the concurrent secondary-outcome models. Panel B presents between-person estimates from the lagged models and additionally adjusts for the corresponding day-*t* CGM outcome, as described in Section 2.9.4. Panel C presents between-person estimates for the secondary exploratory movement exposures described in Section 2.9.5. All between-person estimates were treated as exploratory; interpretation therefore emphasizes effect estimates and 95% confidence intervals rather than confirmatory hypothesis testing. CI: confidence interval; CGM: continuous glucose monitoring; CV: coefficient of variation; MVPA: moderate-to-vigorous physical activity; SD: standard deviation.
