# Supplementary material for: Accelerometer-Derived Physical Activity, Sedentary Behavior, and Continuous Glucose Monitoring-Derived Glycemic Variability in Adults with Type 2 Diabetes: A Longitudinal Repeated-Measures Study
Source: J Funct Morphol Kinesiol. 2026 Aug 28;11(3):338. doi: 10.3390/jfmk11030338 (PMC13608073; doi:10.3390/jfmk11030338)
Supplement: Supplementary file 1 [file jfmk-11-00338-s001.zip › Supplementary Table S3. Lagged associations between.pdf]

Supplementary Table S3. Lagged associations between day *t* movement behaviors and day *t* + 1 CGM-derived outcomes, adjusted for the corresponding day *t* glycemic outcome.

Panel A. Linear mixed-effects lagged models

Exposure on day *t*	Outcome on day *t* + 1	β coefficient	95% CI	Nominal p value	FDR q value
MVPA, per 10 min/day	Coefficient of variation, %	−0.16	−0.31 to −0.01	0.037	0.061
MVPA, per 10 min/day	Time in range, %	0.43	0.04 to 0.82	0.031	0.061
MVPA, per 10 min/day	Time above range, %	−0.40	−0.79 to −0.01	0.044	0.061
MVPA, per 10 min/day	Time below range, %	−0.02	−0.09 to 0.05	0.581	0.581
Accelerometer-defined sedentary time, per 10 min/day	Coefficient of variation, %	0.03	0.00 to 0.06	0.046	0.061
Accelerometer-defined sedentary time, per 10 min/day	Time in range, %	−0.09	−0.17 to −0.01	0.032	0.061
Accelerometer-defined sedentary time, per 10 min/day	Time above range, %	0.09	0.01 to 0.17	0.035	0.061
Accelerometer-defined sedentary time, per 10 min/day	Time below range, %	0.01	−0.01 to 0.03	0.421	0.481

Panel B. Complementary generalized lagged analyses for bounded CGM-derived percentage outcomes

Outcome on day *t* + 1	Exposure on day *t*	Model	Effect measure	Estimate	95% CI	p value
Time in range	MVPA, per 10 min/day	Fractional-logit GEE	Average marginal effect, percentage points	0.41	0.03 to 0.79	0.035
Time above range	MVPA, per 10 min/day	Fractional-logit GEE	Average marginal effect, percentage points	−0.38	−0.75 to −0.01	0.045
Time in range	Accelerometer-defined sedentary time, per 10 min/day	Fractional-logit GEE	Average marginal effect, percentage points	−0.08	−0.16 to −0.01	0.041
Time above range	Accelerometer-defined sedentary time, per 10 min/day	Fractional-logit GEE	Average marginal effect, percentage points	0.08	0.01 to 0.16	0.039
Any TBR (>0% vs. 0%)	MVPA, per 10 min/day	Binomial-logit GEE	Odds ratio	0.98	0.93 to 1.04	0.493
TBR magnitude among TBR >0% days	MVPA, per 10 min/day	Fractional-logit GEE	Average marginal effect, percentage points	−0.02	−0.07 to 0.03	0.439
Any TBR (>0% vs. 0%)	Accelerometer-defined sedentary time, per 10 min/day	Binomial-logit GEE	Odds ratio	1.01	0.99 to 1.03	0.382
TBR magnitude among TBR >0% days	Accelerometer-defined sedentary time, per 10 min/day	Fractional-logit GEE	Average marginal effect, percentage points	0.01	−0.01 to 0.03	0.447

Note: The lagged analyses included 132 participants contributing 1692 valid day-*t* to day-*t* + 1 participant-day pairs. Panel A reports within-person associations per 10 min/day from the temporally ordered models described in Section 2.9.4. These models adjusted for the corresponding CGM outcome on day *t*, decomposed into within-person and between-person components, and retained the fully adjusted Model 3 clinical covariate set described in Section 2.9.2. Benjamini–Hochberg FDR correction was applied across the eight lagged exposure–outcome tests reported in Panel A. Panel B presents complementary generalized analyses for the bounded TIR, TAR, and TBR outcomes. Because 931 of 1692 (55.0%) next-day observations had TBR = 0%, TBR was evaluated using a two-part model. Average marginal effects are expressed in percentage points. CI: confidence interval; CGM: continuous glucose monitoring; FDR: false discovery rate; GEE: generalized estimating equations; MVPA: moderate-to-vigorous physical activity; OR: odds ratio; TAR: time above range; TBR: time below range; TIR: time in range.
