# Supplementary material for: Accelerometer-Derived Physical Activity, Sedentary Behavior, and Continuous Glucose Monitoring-Derived Glycemic Variability in Adults with Type 2 Diabetes: A Longitudinal Repeated-Measures Study
Source: J Funct Morphol Kinesiol. 2026 Aug 28;11(3):338. doi: 10.3390/jfmk11030338 (PMC13608073; doi:10.3390/jfmk11030338)
Supplement: Supplementary file 1 [file jfmk-11-00338-s001.zip › Supplementary Table S4. Sensitivity analyses for the primary mutually.pdf]

Supplementary Table S4. Sensitivity analyses of the primary movement-time model and exploratory secondary-exposure associations with daily 24-h CGM-derived coefficient of variation.

Panel A. Sensitivity analyses of the mutually adjusted movement-time model

Sensitivity analysis	Analytical sample	MVPA β per 10 min/day	95% CI	p value	Accelerometer-defined sedentary time β per 10 min/day	95% CI	p value
Primary mutually adjusted model	132 participants; 1728 participant-days	−0.27	−0.45 to −0.09	0.003	0.05	0.01 to 0.09	0.014
Fully adjusted model excluding HbA1c	132 participants; 1728 participant-days	−0.28	−0.46 to −0.10	0.002	0.05	0.01 to 0.09	0.015
Excluding <12 valid accelerometer days	126 participants; 1704 participant-days	−0.26	−0.44 to −0.08	0.005	0.05	0.01 to 0.09	0.018
Excluding participant-days with <80% CGM data	129 participants; 1676 participant-days	−0.25	−0.43 to −0.07	0.007	0.05	0.01 to 0.09	0.021
Excluding insulin-treated participants	84 participants; 1096 participant-days	−0.22	−0.42 to −0.02	0.031	0.04	0.01 to 0.08	0.033
Additional adjustment for weekend vs. weekday	132 participants; 1728 participant-days	−0.27	−0.45 to −0.09	0.003	0.05	0.01 to 0.09	0.015
Diary-informed time-varying confounding analysis	128 participants; 1562 participant-days	−0.25	−0.43 to −0.07	0.007	0.05	0.01 to 0.09	0.02
AR(1) residual correlation model	132 participants; 1728 participant-days	−0.26	−0.44 to −0.08	0.005	0.05	0.01 to 0.09	0.017
Generalized estimating equations	132 participants; 1728 participant-days	−0.26	−0.46 to −0.06	0.011	0.05	0.01 to 0.10	0.019
Single-exposure models (movement exposures modeled separately)	132 participants; 1728 participant-days	−0.31	−0.49 to −0.13	0.001	0.06	0.02 to 0.10	0.002

Panel B. Sensitivity analysis for alternative MVPA intensity classification

MVPA classification	Analytical sample	β per 10 min/day	95% CI	p value
Primary Freedson threshold (≥1952 counts/min)	132 participants; 1728 participant-days	−0.27	−0.45 to −0.09	0.003
Alternative older-adult calibration threshold (≥1041 counts/min)	132 participants; 1728 participant-days	−0.23	−0.39 to −0.07	0.005

Panel C. Serial-correlation diagnostics for the primary movement-time model

Model specification	Lag-1 residual autocorrelation	AR(1) ρ	AIC	BIC
Participant-specific random intercept	0.17	—	11,846.20	11,962.50
Participant-specific random intercept + AR(1)	0.02	0.16	11,828.90	11,951.40

Panel D. Exploratory within-person associations of secondary accelerometer-derived exposures

Secondary exploratory exposure	Analytical sample	Exposure increment	β coefficient	95% CI	Nominal p value
Daily step count	132 participants; 1728 participant-days	1000 steps/day	−0.18	−0.31 to −0.05	0.007
Light physical activity	132 participants; 1728 participant-days	30 min/day	−0.10	−0.23 to 0.03	0.132
Accelerometer-defined sedentary breaks	132 participants; 1728 participant-days	10 breaks/day	−0.14	−0.29 to 0.01	0.067

Note: Panel A summarizes sensitivity analyses of the primary mutually adjusted movement-time model. Unless otherwise indicated, these analyses retained the within-person and between-person components of MVPA, accelerometer-defined sedentary time, and valid accelerometer wear time, together with the fully adjusted Model 3 clinical covariate set described in Section 2.9.2. The diary-informed analysis additionally adjusted for daily sleep duration and excluded participant-days with documented acute illness or medication changes. Single-exposure models included MVPA and accelerometer-defined sedentary time in separate model specifications. Panel B compares the primary Freedson MVPA classification (≥1952 counts/min) with the alternative ≥1041 counts/min sensitivity classification. Panel C presents residual serial-correlation and model-fit diagnostics for the primary movement-time model. Panel D presents exploratory within-person associations of daily step count, light physical activity, and accelerometer-defined sedentary breaks, as described in Section 2.9.5; the sedentary-break model additionally accounted for daily valid accelerometer wear time. AIC: Akaike information criterion; AR(1): first-order autoregressive correlation structure; BIC: Bayesian information criterion; CI: confidence interval; GEE: generalized estimating equations; MVPA: moderate-to-vigorous physical activity.
